# Mediterranean diet adherence is associated with mitochondrial microproteins Humanin and SHMOOSE; potential role of the Humanin–Nox2 interaction in cardioprotection

**DOI:** 10.3389/fnut.2025.1727012

**Published:** 2026-03-10

**Authors:** Roberto Vicinanza, Vittoria Cammisotto, Junxiang Wan, Kelvin Yen, Francesco Violi, Pasquale Pignatelli, Pinchas Cohen

**Affiliations:** 1 Leonard Davis School of Gerontology, University of Southern California, Los Angeles, CA, United States; 2 Department of Medical and Cardiovascular Sciences, Sapienza University of Rome, Rome, Italy

**Keywords:** cardioprotection, healthspan, Humanin, Mediterranean diet, mitochondrial microproteins, Nox2, oxidative stress, SHMOOSE

## Abstract

**Background:**

The health benefits of the Mediterranean Diet (Med-Diet) have been demonstrated in observational studies and randomized controlled trials. Emerging evidence suggests that the biological effects of the Med-Diet may be mediated by the modulation of mitochondrial function. Human mitochondrial DNA (mtDNA) encodes microproteins, which have been shown to regulate aging, cardiometabolic functions, and neuroprotection.

**Objectives:**

To investigate Humanin and SHMOOSE (Small Human Mitochondrial ORF Over SErine tRNA), as potential mitochondrial biomarkers of Med-Diet adherence and their associations with markers of oxidative stress.

**Methods:**

Cross-sectional analysis of 49 patients (mean age 78.4 ± 8.7 years; 57% female) selected from an observational study of non-valvular atrial fibrillation (AF) conducted at the Atherothrombosis Center of Sapienza University of Rome. Patients were categorized into low-medium (0–6) and high (7–9) adherence to the Med-Diet based on the 9-item Med-Diet questionnaire. Oxidative stress was evaluated by measuring soluble Nox2-derived peptide (sNox2-dp) and plasma 8-iso-prostaglandin F2α (8-iso-PGF2α) using enzyme-linked immunosorbent assays (ELISA). Circulating Humanin and SHMOOSE levels were measured using an in-house sandwich ELISA.

**Results:**

High Med-Diet adherence was observed in 20 patients (40.8%), while 29 patients (59.2%) had low-medium adherence. Patients with high adherence exhibited higher plasma levels of SHMOOSE (*p* = 0.046) and Humanin (*p* = 0.045). The analysis of the dietary components of the Med-Diet revealed higher levels of SHMOOSE with olive oil consumption (*p* = 0.020) and low intake of refined bread (*p* = 0.029), while Humanin positively correlated with olive oil (*p* = 0.0069), fish (*p* = 0.038), and legumes (*p* = 0.0282). Additionally, Humanin was inversely associated with sNox2-dp (*p* = 0.019), which remained significant after adjusting for sex and BMI (*B* = −0.010; *β* = −0.302; *p* = 0.040), and 8-iso-PGF2α (*p =* 0.049).

**Conclusion:**

This study indicates (i) a positive association between adherence to the Med-Diet and circulating levels of mitochondrial microproteins SHMOOSE and Humanin supporting their role as potential mediators of Med-Diet benefits; (ii) a putative crosstalk between Humanin signaling and Nox2 activity, suggesting a novel cardioprotective mechanism of the Med-Diet. Collectively, these findings support mitochondrial microproteins as promising biomarkers for tailoring nutritional strategies for healthy aging. Further studies are warranted to elucidate the underlying mechanisms and determine the causal nature of these associations.

## 1 Introduction

**Figure 1:**
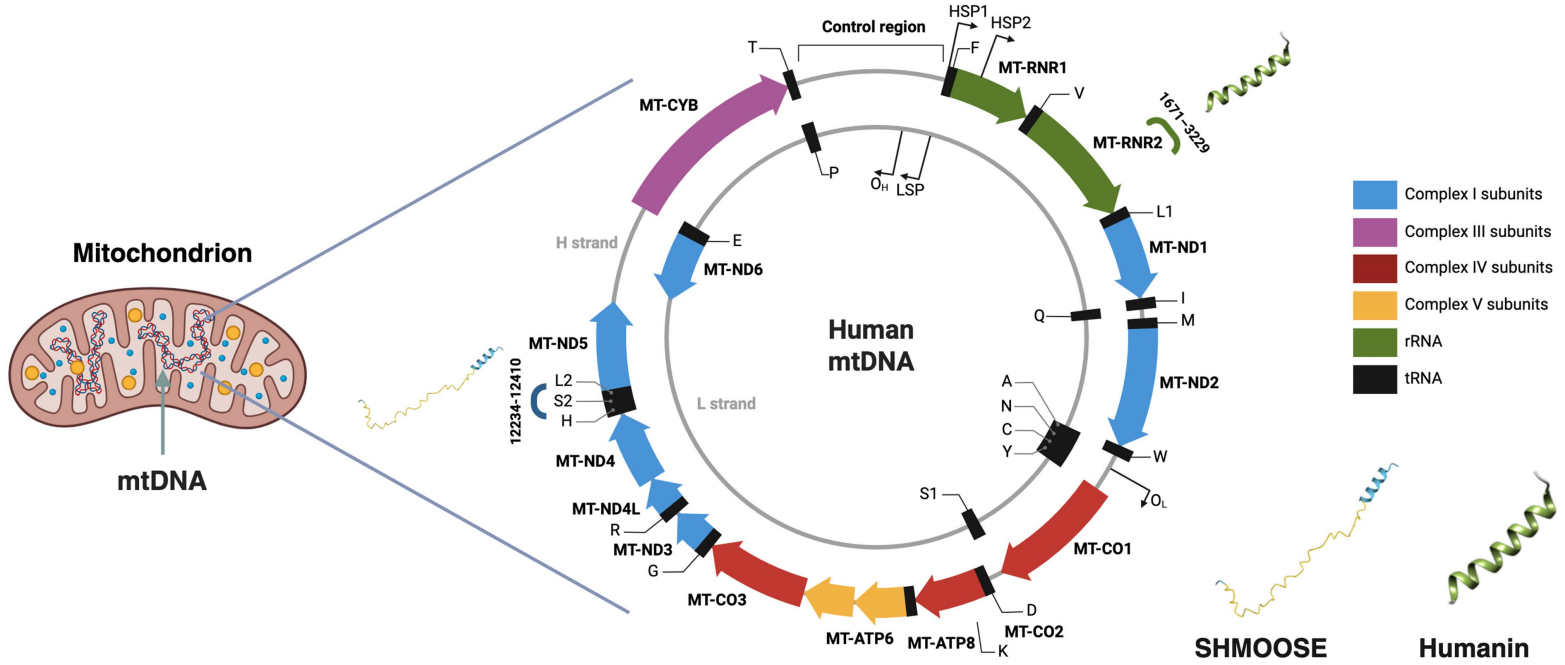
Localization of SHMOOSE and Humanin within the human mitochondrial genome. Schematic representation of the human mitochondrial DNA (mtDNA). The outer circle represents the H-strand, containing most of the mitochondrial genes; the inner circle represents the L-strand. Humanin is encoded within the *MT-RNR2* gene (16S rRNA) between nucleotides 1,671 and 3,229, and SHMOOSE is encoded by a small open reading frame (smORF) overlapping the serine tRNA region, between nucleotides 12,234 and 12,410. The 3D structures of Humanin and SHMOOSE, predicted with AlphaFold 3, are shown. Created in BioRender.

The Mediterranean Diet (Med-Diet) is characterized by a wide variety of nutrient-dense foods, including (i) whole grains, legumes, fruits, and vegetables as the primary sources of carbohydrate, protein and fiber; (ii) nuts and extra virgin olive oil (EVOO), which provide the main dietary fats; and (iii) moderate amounts of fish, poultry, and dairy products, with limited red meat consumption, contributing to overall protein intake (1–3). The Med-Diet is also rich in bioactive polyphenols (also known as phytonutrients), with antioxidant and anti-inflammatory properties (4–6), as well as mono- and polyunsaturated fatty acids, primarily from nuts, fish, and EVOO, that have been shown to improve lipid profile (7, 8) and reduce inflammatory biomarkers (9–11). Observational studies and randomized controlled trials demonstrated that adherence to the Med-Diet may reduce the risk of cardiometabolic disorders, the burden of multimorbidity (12–16) and lower the risk for all-cause mortality (17, 18), thereby promoting healthspan and longevity (19, 20). Emerging evidence suggest that the benefits of the Med-Diet may be mediated, at least in part, by the reduction of oxidative stress (21), as evidenced by decreased activity of NADPH oxidase 2 (Nox2), a major enzymatic source of reactive oxygen species (ROS) (22, 23), and the modulation of the hallmarks of aging (24) including attenuation of mitochondrial dysfunction (25). Mitochondria play a pivotal role in macronutrient metabolism, redox homeostasis, and cellular signaling, and their dysfunction is involved in the pathophysiology of age-related diseases (26). Mitochondria contain circular, double-stranded DNA (mtDNA), which comprises 16,569 base pairs and encodes 37 genes, including protein-coding genes for components of the electron transport chain that drive oxidative phosphorylation, generating most of the cellular energy as adenosine triphosphate (ATP) (27). However, recent research has identified previously unannotated mitochondrial microproteins (also known as mitochondrial-derived peptides, MDPs) encoded by small open reading frames (smORFs) within the mtDNA, which were largely overlooked during the Human Genome Project (28). The term smORF refers to ORFs with less than 100 codons that are actually translated, while the term “microprotein” refers to biologically active proteins shorter than 100 amino acids encoded by the smORFs (29) (Figure 1). Mitochondrial microproteins may be involved in crucial biological functions, including the pathophysiology of age-related diseases and the mechanisms of longevity (30, 31). For example, a recently identified genetic variant of the novel microprotein SHMOOSE (Small Human Mitochondrial ORF Over SErine tRNA) has been shown to mitigate amyloid-β42–induced neurotoxicity, while its genetic variant has been associated with increased risk for Alzheimer’s disease (AD) suggesting neuroprotective effects (32). Similarly, Humanin, a microprotein encoded within the 16S ribosomal RNA region and widely distributed across tissues (33), was discovered by three independent laboratories over 20 years ago for its neuroprotective properties (34–36). More recently, analysis of samples from the Albert Einstein Longevity Genes Project identified a Humanin genetic variant enriched in centenarians, which has been shown to mitigate AD-related amyloid-*β* pathology (37). In addition to its neuroprotective effects, Humanin has been detected in human vascular walls, demonstrating a cytoprotective effects against oxidized LDL-induced oxidative stress in age-related cardiovascular disease (CVD)(38, 40). Moreover, Humanin has been shown to preserve coronary endothelial function (77), and low circulating levels of Humanin have been identified as an independent risk factor for coronary artery disease (CAD) (39). Given the pivotal role of mitochondria in nutrient metabolism and the previously described role of Humanin in cellular stress response (41), it is plausible that the expression of mitochondrial microproteins may be influenced by specific nutrients and dietary patterns. Therefore, based on the evidence linking the adherence to the Med-Diet with reduced oxidative stress, and given the previously reported cardio-and neuroprotective roles of Humanin and SHMOOSE, the present study aimed to examine circulating levels of these microproteins in relation to Med-Diet adherence and to investigate their association with two complementary CVD -related oxidative stress biomarkers, Nox2-derived peptide (sNox2-dp) and 8-iso-prostaglandin F2α (8-iso-PGF2α).

## 2 Study design and methods

### 2.1 Patients recruitment

The present study involved participants from a prospective single-center study of nonvalvular AF referred to the Atherothrombosis Center for evaluation and management of antithrombotic therapies at the Department of Clinical Internal, Anesthesiologic and Cardiovascular Sciences, Policlinico I University Hospital at Sapienza University of Rome. In the initial enrolment, all patients with non-valvular AF, aged >18 years of both sexes were included in the study. Patients were then excluded if they had mechanical or biologic prosthetic valves, severe valvulopathies or history of valvulopasty, congenital heart diseases, severe cognitive impairment, chronic inflammatory diseases, active neoplastic diseases, liver cirrhosis, or if they were taking any nutritional supplements. At baseline, adherence to the Med-Diet was assessed by a physician or registered nurse using the validated 9-item dietary questionnaire (42), and the patients were asked to respond based on their usual eating habits at home.

### 2.2 Selection of the sub-cohort for the evaluation of mitochondrial microproteins

For this cross-sectional pilot study, we selected a convenience sub-cohort of 49 patients from a larger prospective registry. Eligible patients were those who: (i) met the initial inclusion criteria, (ii) had completed the Med-Diet questionnaire, (iii) were aged ≥60 years, and (iv) had sufficient stored plasma available for the measurement of at least two types of mitochondrial microproteins. Patients were categorized into two groups: low-medium adherence (0–6 points) and high adherence (7–9 points). The two groups were further balanced for age, BMI, chronic conditions, and cardiovascular risk factors. Patients with a body mass index (BMI) > 30 kg/m^2^, stage 3B of chronic kidney disease (CKD), or with any acute medical condition at the time of the initial recruitment were excluded. All participants provided written informed consent, and the study protocol was approved by the local ethics committee of Sapienza University of Rome (Rif. 1,306/2007, Prot. 417/14) and conducted in accordance with the principles of the Declaration of Helsinki.

### 2.3 Adherence to Mediterranean diet questionnaire

Adherence to the Mediterranean Diet was assessed using a validated 9-item questionnaire that captures key components of the traditional Med-Diet pattern. The total Med-Diet score ranged from 0 to 9 points. One point was assigned for meeting each of the following criteria: (1) Olive oil as main culinary fat (≥1 tablespoon/day); (2) Fruit (≥1 serving/day); (3) Vegetables or salad (≥1 serving/day); (4) Fruit (≥1 serving/day) and vegetables (≥1 serving/day); (5) Legumes (≥2 servings/week); (6) Fish (≥3 servings/week); (7) Wine (≥1 glass/day); (8) Meat (<1 serving/day); (9) [White bread (<1/day) and rice (<1/week)] or whole-grain bread (>5/week).

### 2.4 Blood sample collections

Fasting blood samples were collected between 8:00 and 9:00 a.m. via venipuncture at the Atherothrombosis Center. Samples were sent to the local laboratories for routine blood analysis, including glucose, lipid profile, liver enzymes, and parameters of kidney function. For the measurement of oxidative stress biomarkers, additional blood samples were collected in 3.8% sodium citrate tubes (1:10 v/v ratio). Samples were then centrifuged at room temperature (RT) for 10 min at 300 g to obtain plasma samples. Plasma was then aliquoted and stored at −80 °C until analysis of Nox2-derived peptide (sNox2-dp) and isoprostanes was performed. Aliquots of the stored samples were later shipped in dry ice to the laboratories of the USC Leonard Davis School of Gerontology (Los Angeles, CA) for the quantification of mitochondrial microproteins, Humanin and SHMOOSE. All samples were labeled with study codes, and the receiving laboratory was blinded to group allocation.

### 2.5 Markers of oxidative stress

Plasma Nox2 levels were measured as sNox2-dp with an Enzyme-Linked Immunosorbent Assay (ELISA) method as previously reported (43). Briefly, the peptide was recognized by binding to a specific monoclonal antibody against the amino acid sequence (224–268) corresponding to the extracellular membrane part of Nox2 (catalytic core of NADPH oxidase), which is released following platelet activation. The enzyme activity was measured spectrophotometrically by the increased absorbance at 450 nm. Values were expressed as pg./mL; intra-assay and inter-assay coefficients of variation were 8.95 and 9.01%, respectively.

Plasma isoprostanes (8-iso-PGF2α) were measured by enzyme immunoassay technology (DRG Inter-national, Springfield, NJ, United States) the values were expressed in pmol/L. Intra-assay and inter-assay coefficients of variation were 5.8 and 5.0%, respectively.

### 2.6 Mitochondrial microprotein assay using the humanin and SHMOOSE ELISA

Plasma levels of Humanin and SHMOOSE were measured using an in-house ELISA (32, 44). Prior to the assay, Humanin and SHMOOSE were extracted from plasma in 90% acetonitrile and 10% 1 N HCl. Briefly, 200 μL of extraction reagent was added to 100 μL of plasma, gently mixed and incubated at room temperature for 30 min. The mixtures were centrifuged, and the supernatants were removed and dried. The dried extracts were reconstituted with 250 mL of phosphate buffer (50 mM sodium phosphate, 150 mM sodium chloride, 0.5% Tween-20, pH 7.6). To measure endogenous Humanin and SHMOOSE levels, synthetic Humanin, and SHMOOSE-c peptides were used as a standard with a range of 50 pg./mL to 10,000 pg./mL. Ninety-six-well microtiter plates were coated with rabbit anti- Humanin or SHMOOSE antibodies at 0.5 mg/well in 200 μL of 50 mM sodium bicarbonate buffer and incubated at room temperature for 3–4 h on a shaker followed by 2 washes with wash buffer and two washes with Superblock buffer (Pierce Chemicals, Rockford, IL). 100 μL of standards, controls or extracted samples and 100 μL of pre-titered biotinylated anti- Humanin or SHMOOSE antibodies were added to the appropriate wells and incubated overnight. Streptavidin-HRP conjugate was added to the wells after washing and further incubated for 30 min at room temperature. After 4 washes with wash buffer, 200 μL/well of 1-step ultra TMB were added and incubated for 10–20 min. The reaction was stopped by the addition of 2 N H2SO4 and absorbance was measured on a plate spectrophotometer (Molecular Designs, Sunnyvale, CA) at 450 nm.

## 3 Statistical analysis and figures preparation

All statistical analyses were performed using SPSS software (version 29.0.2; IBM, Armonk, NY, United States), while graphical representations and figures were generated using GraphPad Prism (version 10.4.1 for Mac, GraphPad Software, Boston, Massachusetts, United States) and BioRender Scientific Image and Illustration Software. The 3D models for Humanin and SHMOOSE were created using Alphafold 3 (45). Descriptive statistics were used to summarize the data, with categorical variables reported as frequencies and continuous variables presented as means ± standard deviation (SD). The Shapiro–Wilk test was used to assess normality. Categorical variables were compared using the Chi-square test, while continuous variables were analyzed using two-tailed independent-samples *t* tests. Variables with non-normal distributions were log-transformed prior to analysis when appropriate. Pearson’s correlation was used to assess associations between variables. Linear regression analyses adjusted for sex and BMI were performed to evaluate independent associations between mitochondrial microproteins and oxidative stress markers. A *p* value < 0.05 was considered statistically significant.

## 4 Results

### 4.1 Patient characteristics

The analysis consisted of 49 patients (57% women). The prevalence of hypertension was 85.7%, while 12.2% had a history of coronary heart disease. History of cerebrovascular disease (CeVD) was present in 12.2% of participants, and 6.1% had chronic obstructive pulmonary disease (COPD), while history of heart failure was 4.1%. The mean age of the participants (±SD) was 78.4 ± 8.7 years, with a mean BMI (kg/m^2^) of 24 ± 3.2. No significant differences were found between the high adherence and low-medium adherence group in terms of lipid metabolism (total cholesterol, HDL, LDL, triglycerides), liver function (AST, ALT) (23) and kidney function. However, consistent with our previous findings (23), patients with high adherence to the Med-Diet had lower levels of sNox2-dp compared to those with low-medium adherence (high adherence, 18.8 ± 8.97 pg./mL vs. low-medium adherence 25.0 ± 8.4 pg./mL; *p* = 0.018) and lower 8-iso-PGF2α levels (high adherence, 122.2 ± 32.05 pmol/L vs. low-medium adherence, 156.2 ± 29.05 pmol/L; *p* < 0.001). Patient characteristics are summarized in Table 1.

**Table 1:** Patient characteristics and oxidative stress biomarkers according to the Med-Diet adherence.

Parameter	Overall	Low-medium (0–6) (*N* = 29)	High (7–9) (*N* = 20)	*p*-value
Age, years	78.5 ± 8.7	78.2 ± 9.6	78.6 ± 7.4	0.877
Female, n (%)	28 (57)	16 (57)	12 (43)	0.45
Hemoglobin, g/dl	13 ± 1.6	12.8 ± 1.5	13.3 ± 1.8	0.243
Platelets, 10^3^/μl	226 ± 101	219 ± 72	237 1 ± 32	0.557
Creatinine, mg/dl	1.0 ± 0.2	1.0 ± 0.3	1.0 ± 0.2	0.339
GFR (CKD-EPI), ml/min/1.73m^2^	61.2 ± 16.0	64.8 ± 19.58	56.5 ± 7.57	0.079
Body mass index, kg/m^2^	24 ± 3.2	24.03 ± 3.42	23.97 ± 2.95	0.955
Body weight, kg	66.5 ± 10.9	66.52 ± 11.96	66.50 ± 9.69	0.996
Total cholesterol, mg/dl	174.5 ± 38.86	170.19 ± 35.84	180.88 ± 43.25	0.406
HDL, mg/dl	50.1 ± 23.4	44.68 ± 16.82	57.12 ± 28.92	0.100
LDL, mg/dl	102.8 ± 29.5	104.84 ± 32.86	99.96 ± 24.91	0.643
Triglycerides, mg/dl	116.4 ± 55.1	117.97 ± 59.95	114.50 ± 50.02	0.848
GOT/AST (<45 U/L)	26 ± 18.7	23.68 ± 10.83	28.98 ± 25.78	0.359
GPT/ALT (<40 U/L)	23.3 ± 18.4	19.52 ± 12.33	28.74 ± 23.92	0.106
Oxidative stress biomarkers				
sNox2-dp, pg./mL	22.5 ± 9.08	25 ± 8.4	18.8 ± 8.97	**0.018**
8-iso-PGF2α, pmol/L	84.19 ± 34.4	156.2 ± 29.05	122.2 ± 32.05	**< 0.001**

Data are presented as mean ± standard deviation (SD) for continuous variables, and categorical variables are reported as frequencies. Group comparisons were performed using the Chi-square test for categorical variables and independent samples *t*-test (two-tailed) for continuous variables; *p*-values < 0.05 were considered statistically significant and are highlighted in bold. Total cholesterol and triglycerides were available for 39 patients; AST and ALT were available for 44 patients.

### 4.2 Mitochondrial microproteins and adherence to the Mediterranean diet

**Figure 2:**
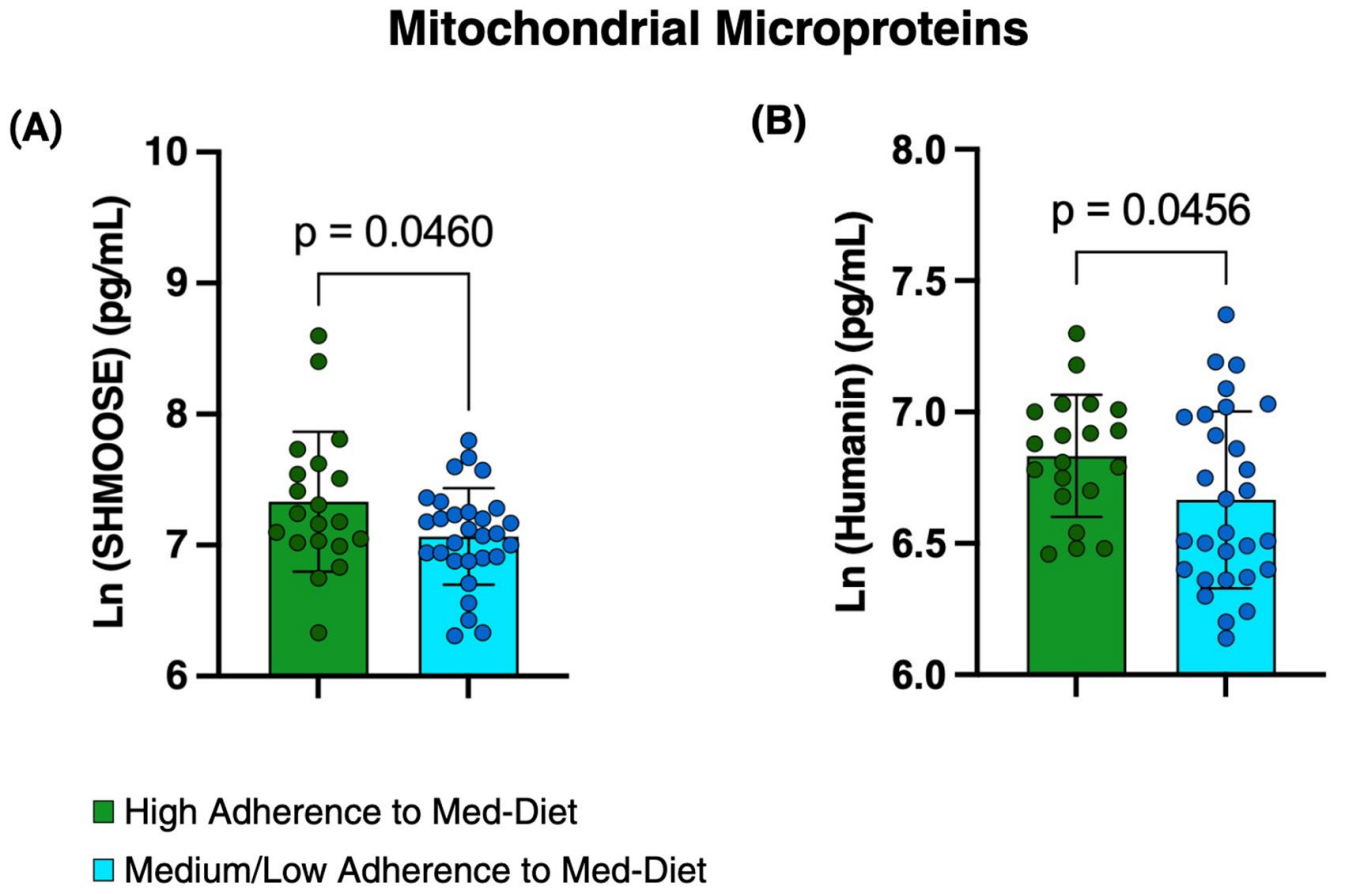
SHMOOSE and Humanin levels according to Med-Diet adherence. Bar graphs represent the mean ± SD of circulating SHMOOSE **(A)** and Humanin **(B)** levels, expressed as natural log-transformed (Ln) values, stratified by high adherence (green) and low-medium adherence (blue) to the Med-Diet. Statistical significance was defined as *p* < 0.05. Created in BioRender.

**Figure 3:**
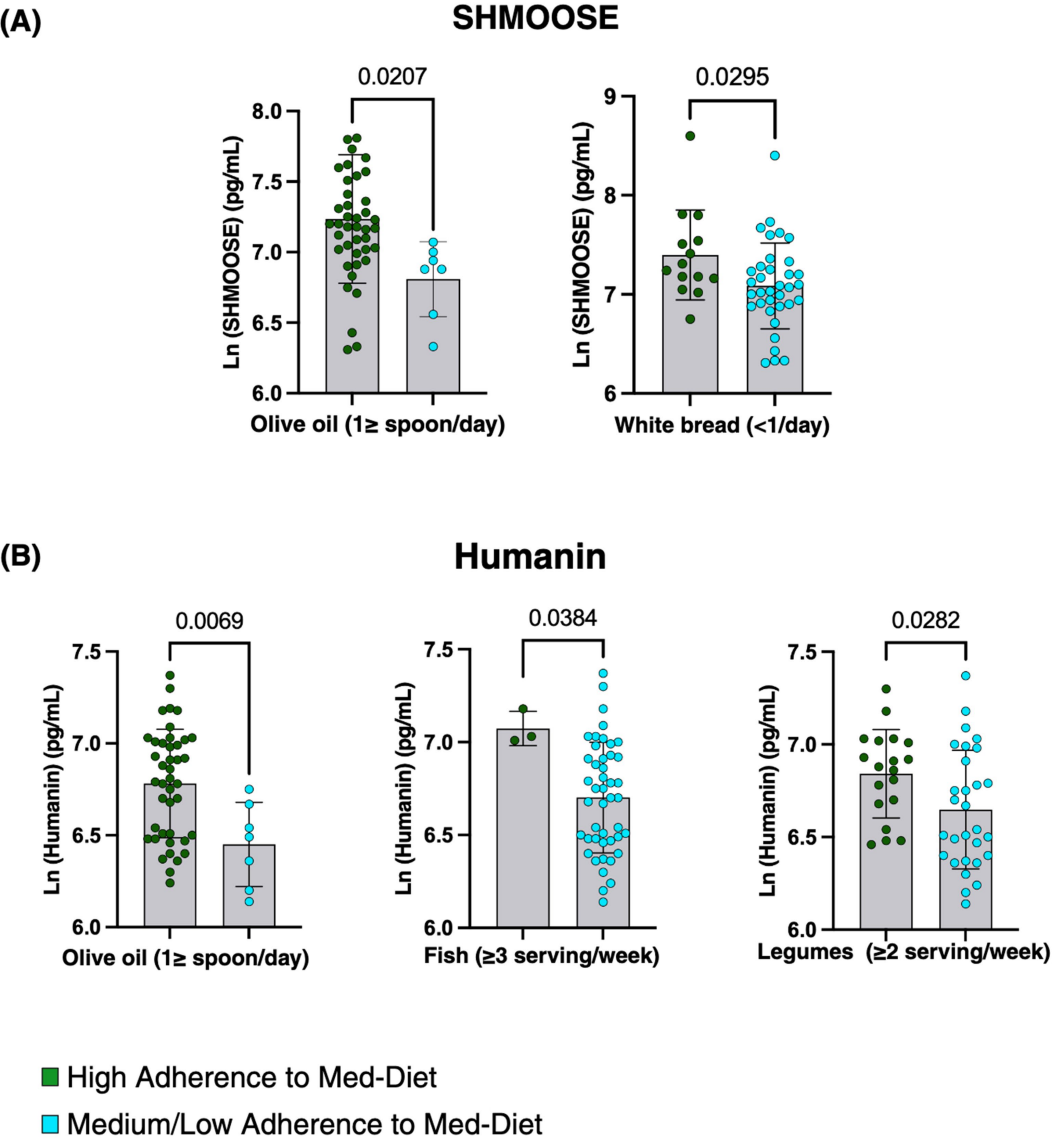
SHMOOSE and Humanin levels according to adherence to the dietary components of the Med-Diet. Bar graphs represent the mean ± SD of circulating SHMOOSE and Humanin levels, expressed as natural log-transformed (Ln) values. **(A)** Higher levels of SHMOOSE with consumption of less than one serving of white bread and at least 1 tablespoon of olive oil per day. **(B)** Higher levels of Humanin with increased consumption of olive oil (≥1 tablespoon/day), fish (≥3 servings/week), and legumes (≥2 servings/week). Green dots represent high adherence to the Med-Diet and blue dots represent low-medium adherence. Statistical significance was defined as *p* < 0.05. Created in BioRender.

The analysis of mitochondrial microproteins, Humanin and SHMOOSE, revealed significant differences between the two groups of Med-Diet adherence. Specifically, SHMOOSE levels were significantly higher in patients with high adherence to the Med-Diet compared with those with low-medium adherence (Ln SHMOOSE: 7.331 ± 0.53 pg./mL vs. 7.067 ± 0.36 pg./mL; *p* = 0.0460). Similarly, Humanin levels were significantly elevated in the high adherence group compared to the low-medium adherence group (Ln Humanin: 6.833 ± 0.23 pg./mL vs. 6.666 ± 0.33 pg./mL; *p* = 0.0456; Figure 2). We further examined the associations between the dietary components of the Med-Diet and plasma levels of mitochondrial microproteins. The analysis revealed the following associations:

(1) SHMOOSE levels were significantly higher in individuals consuming ≥1 tablespoon of olive oil daily (*p* = 0.0207) and in those consuming less than one serving of white bread per day (*p* = 0.0295; Figure 3A).
(2) Humanin levels were significantly higher in individuals consuming ≥1 tablespoon of olive oil daily compared to those with a lower intake (*p* = 0.0069). Additionally, elevated Humanin concentrations were observed in patients consuming ≥3 servings of fish per week (*p* = 0.0384) and ≥2 servings of legumes per week (*p* = 0.0282; Figure 3B).

### 4.3 Relationships between mitochondrial microproteins and oxidative stress biomarkers

**Figure 4:**
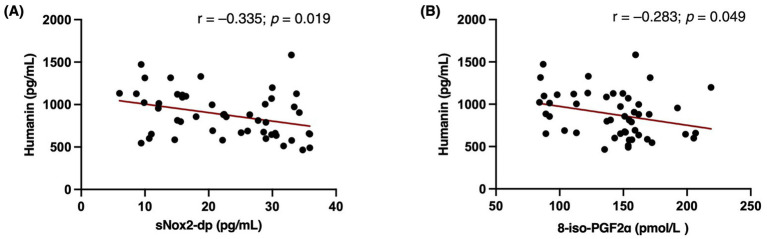
Pearson correlations between circulating Humanin levels and markers of oxidative stress. Scatter plots show a significant inverse association between Humanin and sNOX2-dp (*r* = −0.335; *p* = 0.019, Panel **A**) and between Humanin and 8-iso-PGF2α (*r* = −0.283; *p* = 0.049, Panel **B**). The association between Humanin and sNOX2-dp remained significant after adjustment for age and BMI (B = −0.010; *β* = −0.302; *p* = 0.040). Statistical significance was defined as *p* < 0.05. Created in BioRender.

The analysis of the associations between levels of mitochondrial microprotein and markers of oxidative stress revealed a significant inverse correlation between Humanin and the oxidative stress biomarkers sNOX2-dp (*ρ* = − 0.335; *p* = 0.019) and 8-iso-PGF2α (ρ = − 0.283; *p* = 0.049; Figure 4). However, only the association between Humanin and sNox2-dp remained statistically significant after adjusting for sex and BMI (B = − 0.010; *β* = − 0.302; *p* = 0.040). In contrast, no significant associations were found between SHMOOSE and either sNox2-dp or 8-iso-PGF2α (*p* = 0.346 and *p* = 0.614, respectively).

## 5 Discussion

This study is the first to investigate mitochondrial microproteins, Humanin and SHMOOSE, as potential biomarkers of mitochondrial response to adherence to the Med-Diet, as well as their relationship with the oxidative stress biomarkers sNOX2-dp and 8-iso-PGF2α. Our findings showed that both SHMOOSE and Humanin levels were significantly higher in patients with high adherence to the Med-Diet. Analysis of individual Med-Diet components revealed that SHMOOSE levels were positively associated with olive oil consumption and lower intake of refined bread, whereas Humanin levels were higher in individuals consuming olive oil, fish, and legumes. These findings suggest that circulating levels of mitochondrial microproteins may reflect bioenergetic adaptations to specific nutrient patterns characteristic of the Med-Diet. Furthermore, the inverse correlation between Humanin and sNox2-dp, a direct marker of Nox2 activation (46), indicated a potential interaction between Humanin and Nox2, with Humanin possibly acting as an endogenous modulator of Nox2, thereby contributing to the cardioprotective effects of the Med-Diet (4, 47, 48).

The Med-Diet is recognized as the most evidence-supported dietary pattern for the prevention of cardiometabolic conditions and it is widely recommended in clinical nutrition to improve blood pressure, glycemic control, and lipid profile (49). Additionally, the Med-Diet has been shown to reduce circulating levels of pro-inflammatory cytokines such as tumor necrosis factor-*α* (TNF-α) and interleukin-6 (IL-6) (50, 51), as well as to attenuate oxidative stress via downregulation of Nox2 (23). However, whether mitochondrial microproteins may contribute to the biological effects of the Med-Diet remains largely unexplored. In our study, higher circulating levels of SHMOOSE were associated with lower intake of refined bread. This association may reflect the favorable metabolic effects of consuming minimally processed foods on glycemic control and suggests a possible additional role in supporting mitochondrial homeostasis (52, 53). Indeed, refined carbohydrate may induce intracellular glucotoxicity, leading to excessive ROS production and impaired activity of mitochondrial transcription factor A (TFAM), a central regulator of mtDNA replication and transcription, which could in turn alter mitochondrial microprotein expression (54). Furthermore, the elevated levels of SHMOOSE and Humanin observed among individuals consuming olive oil could be linked to the effects of olive oil–derived polyphenols (e.g., hydroxytyrosol and oleuropein) on SIRT1 activation and PGC-1α deacetylation, which may in turn stimulate mitochondrial biogenesis and, plausibly increasing mitochondrial microprotein levels (55–57). Similarly, the increase in Humanin associated with fish and legumes intake may be explained by the effects of omega-3 polyunsaturated fatty acids (n3-PUFAs) on the AMPK/SIRT1/PGC-1α pathway, and legume-derived polyphenols (e.g., flavonoids, saponins, isoflavones) on the modulation of multiple molecular pathways involved in mitochondrial functions (58–60).

**Figure 5:**
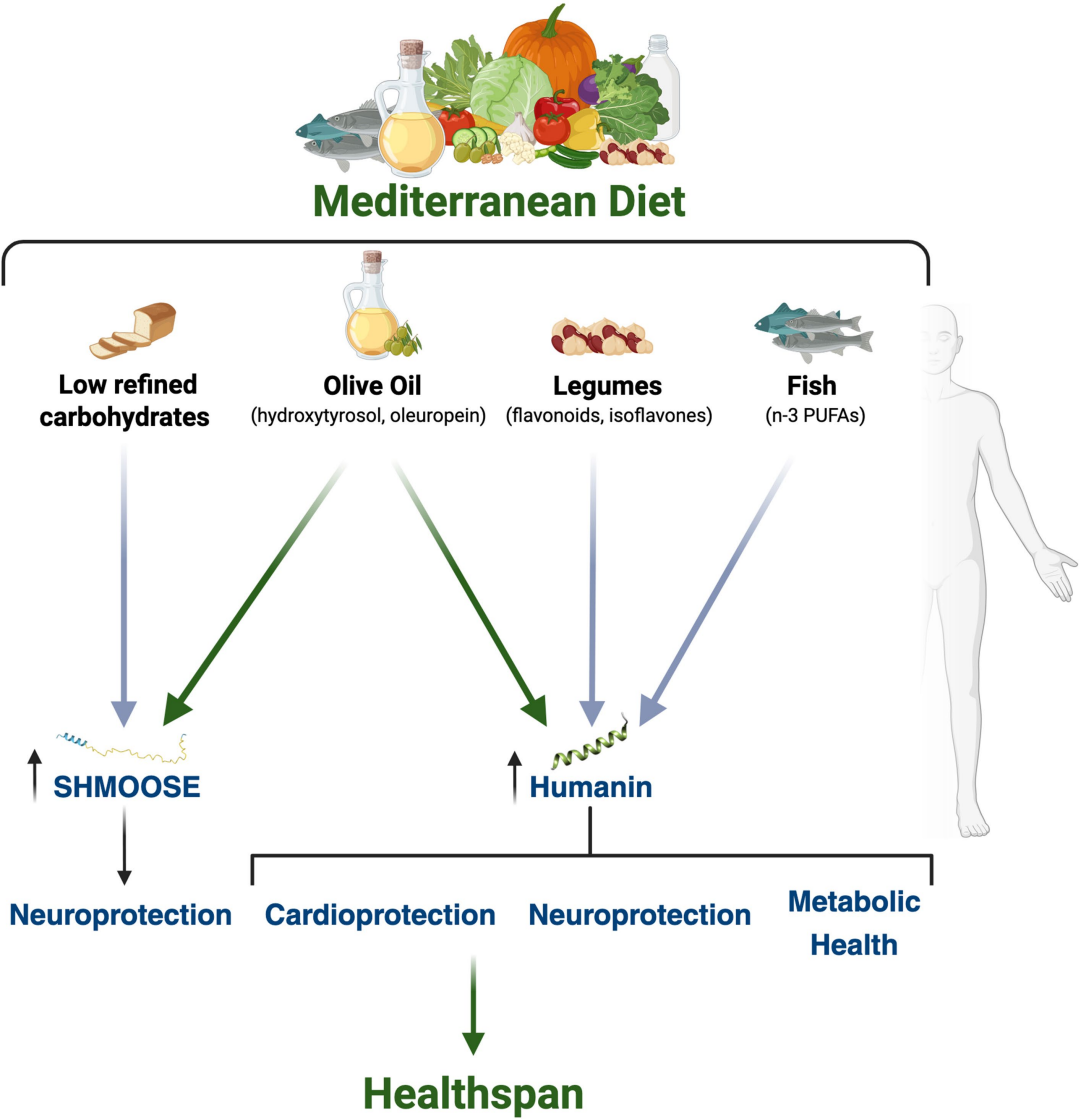
Graphical representation of SHMOOSE and Humanin as potential effectors of Med-Diet–associated benefits. Conceptual diagram illustrating the link between adherence to dietary components of the Med-Diet and its associated neuroprotective, cardioprotective, and metabolic benefits, potentially mediated by increased levels of the mitochondrial microproteins Humanin and SHMOOSE, ultimately promoting healthspan. Created in BioRender.

Although speculative, our results suggest a novel biological trajectory through which mitochondrial microproteins may contribute to the metabolic, cardiovascular, and neuroprotective benefits of the dietary components of the Med-Diet (Figure 5). This hypothesis is supported by accumulating evidence showing that mitochondrial microproteins exert biological effects consistent with those attributed to the Med-Diet. For instance, both Humanin and SHMOOSE have been shown to play a role in AD–related neuropathology by attenuating amyloid-*β*42–induced neurotoxicity (32, 37, 61, 62), aligning with the accumulating evidence linking high adherence to the Med-Diet with favorable cognitive outcome (63–65).

In terms of metabolic effects, both *in vitro* and *in vivo* studies have consistently demonstrated that Humanin may enhance insulin sensitivity and promote pancreatic β-cell survival. Specifically, Humanin inhibited cytokine-induced apoptosis in β-cells and prevented the onset of diabetes in non-obese diabetic mice (66). Additionally, both central (intracerebroventricular) and peripheral administration of Humanin, or its analogs, improved insulin sensitivity and reduced hepatic glucose production via hypothalamic STAT-3 signaling (67). In the clinical setting, circulating Humanin levels were lower in patients with type 2 diabetes mellitus (T2DM) and inversely correlated with HbA1c (68). Notably, these metabolic effects appeared to mimic those elicited by adherence to the Med-Diet (69). Indeed, in the PREDIMED-Reus trial, adherence to the Med-Diet was associated with a 52% reduction in T2DM incidence (70), improved glycemic control (71) lower HbA1c levels (72) and delayed disease progression (73).

Finally, Humanin has also emerged as a cardioprotective factor. In vascular tissue, studies have shown that Humanin may limit oxidized-LDL–induced oxidative stress in endothelial cells (38), and in mice, Humanin preserved endothelial function, prevented the progression of the atherosclerotic plaque (74) and attenuated myocardial ischemia (75). In humans, low circulating Humanin levels have been associated with coronary artery disease, suggesting its role as a potential biomarker for major cardiovascular events (39).

**Figure 6:**
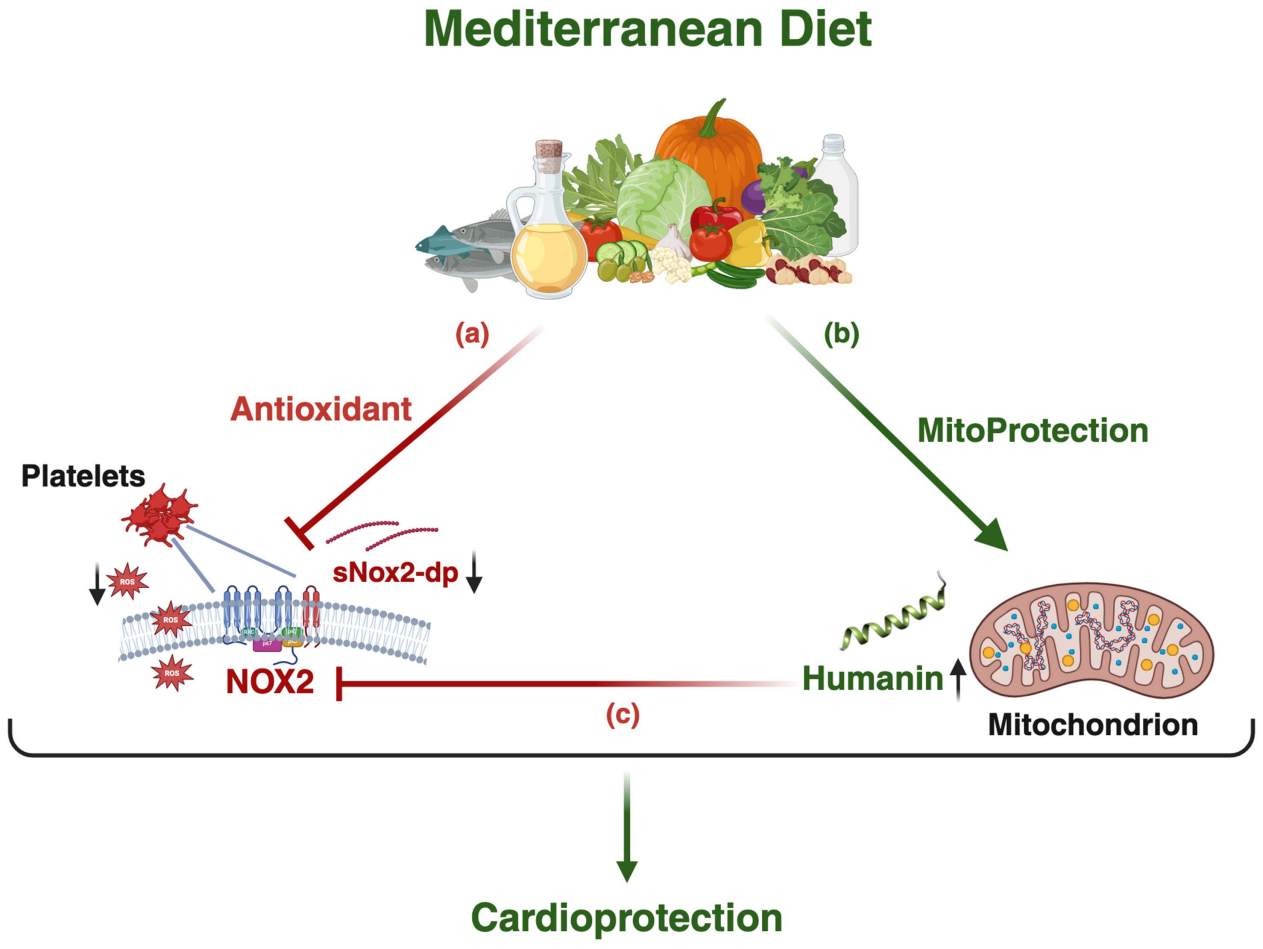
Theoretical model of the cardioprotective effects of the Med-Diet via the Humanin–Nox2 pathways. The schematic model illustrates: **(a)** the antioxidant effects of the Med-Diet, reducing circulating sNox2-dp levels via downregulation of Nox2 activity; **(b)** the mito-protective effects of the Med-Diet resulting in an increase in Humanin levels; and **(c)** the inhibitory effect of Humanin on Nox2 activity, further amplifying the antioxidant and cardioprotective effects of the Med-Diet. Created in BioRender.

In our study, higher Humanin levels were not only associated with greater adherence to the Med-Diet but also with lower levels of oxidative stress biomarkers, particularly sNox2-dp, suggesting a potential molecular crosstalk between Humanin and Nox2. Supporting this hypothesis, Li et al. demonstrated in an *in vitro* model of endothelial free-fatty acid toxicity that Humanin treatment reduced oxidative stress by downregulating Nox2 expression (76), indicating that the inverse correlation between Humanin and sNox2-dp was likely to be causal rather than merely associative. Therefore, as shown in Figure 6, we hypothesize that the lower sNox2-dp observed in patients with high Med-Diet adherence may resulted from both the intrinsic antioxidant properties of the Med-Diet and a potential inhibitory effect of Humanin on Nox2 activity ultimately contributing to cardioprotection. The cardiovascular relevance of this observation is further supported by findings from our previous larger prospective single-center study in patients with AF, in which higher adherence to the Med-Diet, together with lower Nox2 levels, was associated with a reduced incidence of cardiovascular events (23). Taken together, these findings suggest that Humanin may act as a mediator of the cardiovascular benefits of the Med-Diet via Nox2 inhibition, providing a rationale for translational studies to further elucidate the cardioprotective role of the Humanin–Nox2 pathway.

The present study has several limitations. First, its observational design precludes causal inference, and the relatively small sample size, together with the restricted age range, may limit generalizability and reduce the ability to perform robust statistical analyses or stratification by age and sex. Second, the analyzed sub-cohort consisted of individuals with a moderate burden of chronic diseases, which may have contributed to inter-individual variability in mitochondrial microprotein levels. Third, dietary intake was assessed using a brief questionnaire which, although practical and widely used, may not fully capture the complexity of the Med-Diet. Fourth, our study did not account for physical activity levels, metabolic parameters, or other potential confounding factors; therefore, controlled dietary intervention studies are warranted to validate and extend these findings. Finally, the limited availability of stored plasma samples restricted the opportunity to measure additional mitochondrial microproteins. Nevertheless, this pilot study has several strengths: (i) the blinded measurement of mitochondrial microproteins, minimizing measurement bias; (ii) the novel application of mitochondrial microproteins in nutritional research; and (iii) the inverse correlation between Humanin and oxidative stress biomarkers associated with CVD, which strengthens the biological plausibility of the findings.

## 6 Conclusion

This pilot study suggests that: (i) the Med-Diet and its specific components may positively influence mitochondrial function, leading to increased levels of mitochondrial microproteins; (ii) Humanin may modulate Nox2 activity, potentially enhancing the antioxidant and cardioprotective effects of the Med-Diet; and (iii) mitochondrial microproteins may mediate the pro-longevity effects of the Med-Diet, serving as potential biomarkers of mitochondrial response to dietary interventions and opening new perspectives for precision nutrition strategies aimed at promoting healthy aging.

## Data Availability

The raw data supporting the conclusions of this article will be made available by the corresponding author upon reasonable request.
